# Koumine Decreases Astrocyte-Mediated Neuroinflammation and Enhances Autophagy, Contributing to Neuropathic Pain From Chronic Constriction Injury in Rats

**DOI:** 10.3389/fphar.2018.00989

**Published:** 2018-08-30

**Authors:** Gui-lin Jin, Rong-cai Yue, Sai-di He, Li-mian Hong, Ying Xu, Chang-xi Yu

**Affiliations:** ^1^Department of Pharmacology, College of Pharmacy, Fujian Medical University, Fuzhou, China; ^2^Fujian Key Laboratory of Natural Medicine Pharmacology, College of Pharmacy, Fujian Medical University, Fuzhou, China

**Keywords:** koumine, neuropathic pain, astrocyte, autophagy, apoptosis, rats

## Abstract

Koumine, an indole alkaloid, is a major bioactive component of *Gelsemium elegans*. Previous studies have demonstrated that koumine has noticeable anti-inflammatory and analgesic effects in inflammatory and neuropathic pain (NP) models, but the mechanisms involved are not well understood. This study was designed to explore the analgesic effect of koumine on chronic constriction injury (CCI)-induced NP in rats and the underlying mechanisms, including astrocyte autophagy and apoptosis in the spinal cord. Rats with CCI-induced NP were used to evaluate the analgesic and anti-inflammatory effects of koumine. Lipopolysaccharide (LPS)-induced inflammation in rat primary astrocytes was also used to evaluate the anti-inflammatory effect of koumine. We found that repeated treatment with koumine significantly reduced and inhibited CCI-evoked astrocyte activation as well as the levels of pro-inflammatory cytokines. Meanwhile, we found that koumine promoted autophagy in the spinal cord of CCI rats, as reflected by decreases in the LC3-II/I ratio and P62 expression. Double immunofluorescence staining showed a high level of colocalization between LC3 and GFAP-positive glia cells, which could be decreased by koumine. Intrathecal injection of an autophagy inhibitor (chloroquine) reversed the analgesic effect of koumine, as well as the inhibitory effect of koumine on astrocyte activation in the spinal cord. In addition, TUNEL staining suggested that CCI-induced apoptosis was inhibited by koumine, and this inhibition could be abolished by chloroquine. Western blot analysis revealed that koumine significantly increased the level of Bcl-xl while inhibiting Bax expression and decreasing cleaved caspase-3. In addition, we found that koumine could decrease astrocyte-mediated neuroinflammation and enhance autophagy in primary cultured astrocytes. These results suggest that the analgesic effects of koumine on CCI-induced NP may involve inhibition of astrocyte activation and pro-inflammatory cytokine release, which may relate to the promotion of astrocyte autophagy and the inhibition for apoptosis in the spinal cord.

## Introduction

Neuropathic pain (NP) is a severe and intolerable disease and is considered one of the most difficult pain syndromes to treat due to its complex pathogenesis (Gilron et al., 2015). Despite decades of study, the currently available drugs largely fail to control NP.

Koumine, an indole alkaloid isolated from *Gelsemium elegans*, has shown diverse pharmacological actions including antitumor, anti-inflammatory, anxiolytic, and analgesic activity (Jin et al., 2014; Zhang et al., 2015; Chen et al., 2017). We recently reported the analgesic effect of koumine in various animal pain models, such as chronic constriction injury (CCI), spared nerve injury, diabetic NP, and rheumatoid arthritis pain models (Xu et al., 2012; Ling et al., 2014; Qiu et al., 2015; Yang et al., 2016; Xiong et al., 2017; Jin et al., 2018a). Koumine displayed high efficiency and low toxicity in the treatment of NP, implying that this compound may have potential as a new anti-NP drug. However, its analgesic mechanism against NP still needs to be further explored.

The mechanisms of NP are involved in both peripheral and central sensitization, but the precise mechanism is still unclear (Meacham et al., 2017). In recent years, spinal astrocytes have been reported to play a significant role in the induction and maintenance of NP (Mika et al., 2013). Astrocytes, which form close contacts with neuronal synapses, constitute the most abundant cell type in the central nervous system. Under some circumstances, such as peripheral nerve injury, tissue damage, and arthritis, spinal astrocytes rapidly transform to an activated state, which displays a closer correlation with chronic pain behaviors (Gao and Ji, 2010). Activated astrocytes can regulate the release of neurotrophic factors, inflammatory mediators, chemokines, adenosine and neurotransmitters, resulting in long-lasting thermal hyperalgesia and mechanical allodynia (Zhang et al., 2017). Although the pathogenesis of NP has not yet been fully elucidated, an inflammatory response caused by astrocyte activation is considered one of the most critical events. Therefore, decreasing spinal astrocyte activation by using pharmacotherapeutic approaches could be a therapeutic strategy for NP (Gao and Ji, 2010; Svensson and Brodin, 2010).

Autophagy is a process in which cells use lysosomes to degrade their own damaged organelles and macromolecules, which is an important mechanism for cell survival, differentiation, and development (Mizushima and Komatsu, 2011). Under physiological conditions, autophagy is maintained at a low level. Under endoplasmic reticulum stress, however, autophagy is activated as a defense mechanism, playing an important role in maintaining intracellular environmental homeostasis. In recent years, a number of studies have shown that impairment of autophagy plays an important part in the occurrence and development of NP (Jung and Lim, 2015). In contrast, upregulation of autophagy can slow the process of NP (Kosacka et al., 2013; Marinelli et al., 2014; Berliocchi et al., 2015; Huang et al., 2016). Interestingly, relatively few studies have revealed any participation of autophagy in astrocyte functions that are related to CNS diseases such as Alzheimer’s disease, cerebral ischemia, and spinal cord injury (Guo et al., 2014; Goldshmit et al., 2015; Pan et al., 2015). According to a recent report, Schwann cells, the neuroglia found in the peripheral nervous system, are involved in the development and maintenance of NP through regulation of autophagy (Marinelli et al., 2014). All these findings prompted us to explore the analgesic effect of koumine and its molecular mechanism involved in astrocyte autophagy. The mitochondria-mediated pathway is one of the most important signaling pathways in apoptosis, and energy depletion in mitochondria is an early event in CCI rats (Keilhoff et al., 2016). Furthermore, to clarify the protective effects via the mitochondria-mediated apoptosis pathway, were examined the expression levels of related proteins.

We previously described that the attenuation of NP by koumine may result from the inhibition of neuroglial activation and inflammation response. Here, we extended the initial study and investigated the underlying analgesic mechanisms of koumine, focusing on astrocyte activation, autophagy and apoptosis in the spinal cord.

## Materials and Methods

### Animals

Male Sprague-Dawley rats, weighing 150–180 g or born within 24 h of each other, were provided by the Department of Experimental Animal Center, Fujian Medical University. The rats were housed 6–7 per cage and were provided *ad libitum* access to laboratory chow and water. The rodents were maintained at a constant room temperature (25 ± 2°C), with a regular 12:12-h light/dark schedule, with lights on from 08:00 to 20:00. The rats were used for experiments after an acclimation period of 3–7 days. The experimental protocols were approved by the ethics committee at Fujian Medical University (No: 2016-026), and the study was conducted in accordance with the guidelines published in the NIH Guide for the Care and Use of Laboratory Animals.

### Drugs

**FIGURE 1:**
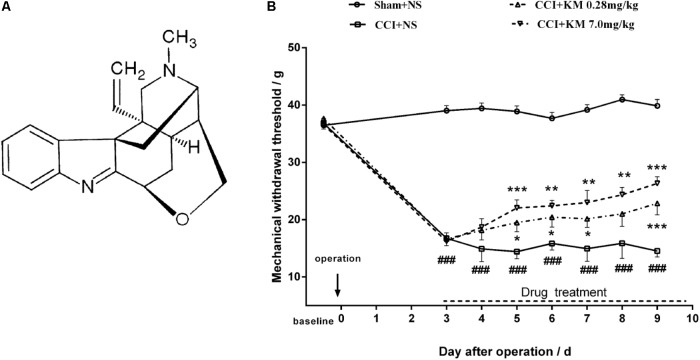
Koumine (KM) displayed an antinociceptive effect. **(A)** Structural formula of koumine. **(B)** Effects of koumine on mechanical allodynia in rats with CCI neuropathy. Rats were conducted Sham or CCI operation. The day of operation was regarded as day 0. Koumine (0.28, 7 mg/kg) or vehicle was administered subcutaneously (s.c.) once per day for seven consecutive days from postoperative day 3. The mechanical withdrawal threshold was measured before surgery (baseline) and drug treatment (predosing), and 1 h after drug administration (postdosing). Data indicate withdrawal threshold for the ipsilateral paw as the mean ± SEM. ^###^*P* < 0.001 versus Sham group. ^∗^*P* < 0.05, ^∗∗^*P* < 0.01, and ^∗∗∗^*P* < 0.001 versus vehicle control group, two-way repeated-measures ANOVA followed by LSD or Dunnett’s T3 test for each time point.

Koumine (PubChem CID: 91895267; purity >98.5%, HPLC; **Figure 1A**) was isolated from *Gelsemium elegans* Benth. via pH-zone-refining countercurrent chromatography, which has been described in our previous study (Su et al., 2011). Koumine was subcutaneously (s.c.) administered at a dose volume of 4 ml/kg dissolved in sterile physiological saline (0.9% NaCl). Chloroquine (CQ), purchased from Sigma (St. Louis, MO, United States), was intrathecally administered at 0.1 μg (20 μL) 15 min after koumine administration.

### CCI Model Preparation

The CCI model was established as previously described (Bennett and Xie, 1988). Briefly, the rats were anesthetized with pentobarbital sodium (40 mg/kg, i.p.), and blunt dissection was performed on the biceps brachii to expose the sciatic nerve. Following separation, the sciatic nerve was ligated with 4.0 silk at 1 mm intervals. A sham group underwent the same surgery, but without ligation. Afterward, the muscle and skin incisions were closed separately.

### Behavioral Testing

The mechanical withdrawal threshold (MWT) was assessed using an electronic von Frey device (series 2390; IITC Life Science Inc., Woodland Hills, CA, United States), as previously described (Mitrirattanakul et al., 2006; Jin et al., 2018a). All tests were performed by a behavioral investigator blinded to the pharmacological treatment of the animals.

### Primary Astrocyte Cultures and Cell Viability Assay

The primary astrocyte cultures and cell viability assay were as previously described (Jin et al., 2018b).

### Western Blot

Rats were anesthetized with sodium pentobarbital after behavioral testing. The L4–L5 spinal segments were quickly isolated and collected in a tissue lysis buffer containing protease inhibitors. Insoluble pellets were separated by centrifugation (14000 × *g* for 30 min, 4°C). The protein samples were quantified by a BCA protein assay kit. The protein samples from the astrocytes were handled as previously described (Jin et al., 2018a). The blot was incubated overnight at 4°C with the primary antibodies mouse polyclonal anti-GAPDH (1:3000, TransGen Biotech, Beijing, China), mouse polyclonal anti-GFAP, rabbit polyclonal anti-LC3B (1:1000, Cell Signaling Technology, Beverly, MA, United States), rabbit polyclonal anti-Beclin 1, rabbit polyclonal anti-p62 (1:1000, MBL International Corporation, Nagoya, Japan), anti-Bcl-xl, anti-Bax, and anti-cleaved caspase-3 (1:1000, Beyotime, Shanghai, China), followed by goat anti-mouse horseradish peroxidase-labeled antibody (1:5000, Jackson Immuno Research Labs Inc., West Grove, PA, United States) or goat anti-rabbit horseradish peroxidase-labeled antibody (1:5000, Jackson Immuno Research Labs Inc., West Grove, PA, United States) for 1 h at room temperature. After these processes, membranes were washed thrice with TBST. Chemiluminescence was detected by using Carestream Molecular Imaging system for 1–5 min. Equal protein loading was confirmed in all the experiments by using GAPDH as loading control. The intensity of each selected band was analyzed using NIH Image J software.

### Immunofluorescence

The lumbar spinal cord segments were removed and postfixed in the fixative overnight. Tissue was then maintained in 30% sucrose in 0.1 M PBS at 4°C overnight. Dissected tissue was mounted in OCT compound and frozen at -20°C. The transverse spinal cord was cut at a thickness of 25 μm in a cryostat (Microm HM 505E). For immunohistochemistry analysis, the sections were washed in 0.01 M PBS 3 times (5 min each) and then blocked with 10% normal goat serum in 0.3% Triton X-100 for 1 h. After being blocked, the sections were incubated overnight at 4°C in the dark with a primary antibody, either anti-GFAP polyclonal antibody or LC3 polyclonal antibody (1:200; Cell Signaling Technology, Beverly, MA, United States). The sections were then washed three times with PBS for 10 min each and incubated with Alexa Fluor 488 anti-mouse antibody (1:200, Jackson Immuno Research Labs Inc., West Grove, PA, United States) and Alexa Fluor 594 anti-rabbit antibody (1:400, Jackson Immuno Research Labs Inc., West Grove, PA, United States) in blocking solution without Triton X-100 for 45 min in the dark. Negative staining controls were prepared by omitting either the primary antibody or secondary antibody. Fluorescent images of these sections were captured with a digital camera (Nikon 80i, Japan), and the fluorescence density was analyzed using a computer software (Image-Pro Plus 6, Media Cybernetics, United States).

### Electron Microscope

Rat spinal cords selected at random from each group were fixed in 4% neutral buffered paraformaldehyde, and then dehydrated for 24 h. After that, spinal cords were embedded in paraffin wax, cut into 3 μm-thick slices, and then examined via electron microscope (Hitachi Co. Ltd., Tokyo, Japan) operated at 75 kV.

### ELISA

After behavioral testing was complete, rat spinal cords were collected, homogenized, and then stored at -80°C. Total TNF-α, IL-6, and IL-1β levels were measured using ELISA Kits (Abcam, ab46070 for TNF-α and ab100768 for IL-1β, Cambridge, MA, United States) according to the manufacturer’s instructions.

### TUNEL Staining

After the rats were sacrificed, the tissues were embedded, sectioned, and deparaffinized. The sections were performed using TUNEL kits according to the manufacturer’s instructions (Beyotime, C1088, Shanghai, China). Sections were observed under a light microscope (Olympus, Tokyo, Japan).

### Statistical Analysis

Data are expressed as the mean ± SEM. Two-way repeated-measures analysis of variance (ANOVA) was used for behavior test. For analysis of immunohistochemistry, ELISA and Western blot analysis, the data were analyzed by one-way ANOVA followed by Dunnett’s *post hoc* test or the least significant difference (LSD) test, respectively. *P*-value <0.05 was defined significant.

## Results

### Koumine Displayed Antinociceptive Effect and Promoted Autophagy in CCI Rats

**FIGURE 2:**
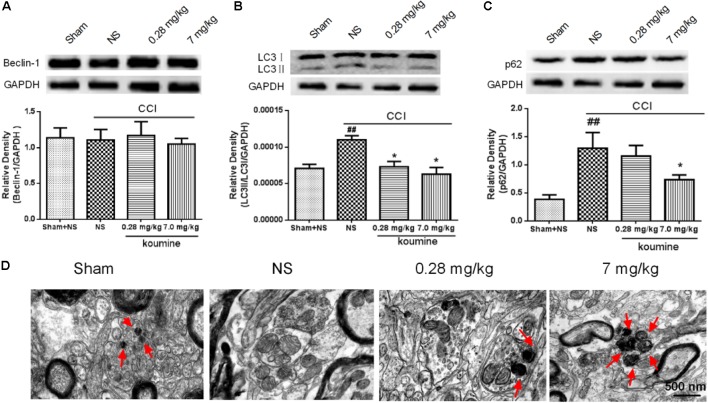
Koumine promoted autophagy in CCI rats. Beclin 1 **(A)**, LC3 **(B)**, and p62 **(C)** levels were detected by Western blot in the spinal cord. **(D)** Spinal cord sections were analyzed by light microscopy electron microscope (scale bar = 500 nm), double layer or multilayer membrane and inclusions are the characteristics of the autophagosome structure which contains mitochondria, endoplasmic reticulum, ribosome, and other cytoplasm components (red arrow). ^##^*P* < 0.05 versus sham group. ^∗^*P* < 0.05 versus vehicle control group by one-way ANOVA followed by LSD test.

Similar to previous studies (Jin et al., 2018a), the present study displayed that repeated subcutaneous administration of KM significantly alleviates NP in CCI rats (**Figure 1B**). To further assess the hypothesis that koumine promotes autophagy in CCI-induced NP rats, we evaluated LC3, Beclin-1, and p62 levels in the rat spinal cord by Western blot. As shown in **Figure 2A**, the level of Beclin-1 showed no remarkable changes among the sham, vehicle-treated CCI and koumine-treated CCI groups. However, a significant increase in the ratio of LC3-II to LC3-I protein was observed after CCI operation in comparison to the sham group (*P* < 0.01). The koumine-treated CCI group further decreased its LC3-II/I ratio compared to vehicle-treated CCI group (*P <* 0.05, **Figure 2B**). SQSTM1/p62 is a critical autophagy protein, and the expression of SQSTM1/p62 may increase when autophagy is impaired. Consistent with the previously study, the protein level of CCI group displayed a significant increase compared to the sham group (*P <* 0.01, **Figure 2C**), and koumine treatment reversed this effect (*P <* 0.05). In addition, transmission electron micrograph showed the characteristics of the autophagosome structure in spinal cord of rats. Double layer or multilayer membrane and inclusions which contains cytoplasm components such as mitochondria, endoplasmic reticulum were displayed by red arrow (**Figure 2D**). Transmission electron micrograph analysis showed an increase in autophagosome formation in the koumine treatment group compared to the CCI group. These results indicate that koumine attenuates NP and alleviates the impaired autophagy in the spinal cord of rats.

### Koumine-Induced Autophagy Occurred in Astrocytes in CCI Rats

**FIGURE 3:**
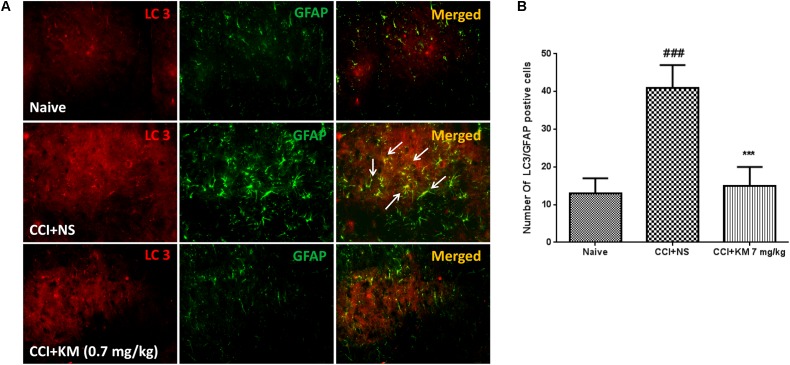
Koumine (KM) inhibit GFAP protein levels in rat spinal cord with CCI neuropathy. **(A)** After CCI or Sham surgery, koumine (0.28, 7 mg/kg) or vehicle was administered s.c. once a day, every day for seven consecutive days from postoperative TSPO and LC3 was determined by Immunofluorescence. **(B)** The number of LC3/GFAP positive cells. The scale bar represents 25 μm. Data are expressed as the mean ± SEM. ^###^*P* < 0.001 versus Sham group. ^∗∗∗^*P* < 0.001 versus vehicle control group, separate one-way ANOVA followed by LSD test.

We previously reported that koumine significantly decreased astrocyte activation as reflected by the specific marker GFAP in spinal cord sections (Jin et al., 2018a). To further investigate whether koumine-induced autophagy occurred in astrocytes, we co-immunostained the spinal cord sections with LC3 and GFAP (**Figure 3A**). In the spinal dorsal horn of the operated side, the LC3 staining showed a high level of colocalization with GFAP-positive glial cells in saline-treated CCI group (*P <* 0.001), while koumine treatment greatly decreased the positive cells of GFAP and LC3, as well as colocalization of LC3 and GFAP (*P <* 0.001, **Figure 3B**). These results indicated that koumine’s alleviation of the impaired autophagy may occur in astrocytes in the spinal cord of CCI rats.

### Koumine Enhances Astrocyte Autophagy and Attenuates Astrocyte Activation, As Well As the Inflammation Response in Lipopolysaccharide (LPS)-Exposed Primary Astrocytes

**FIGURE 4:**
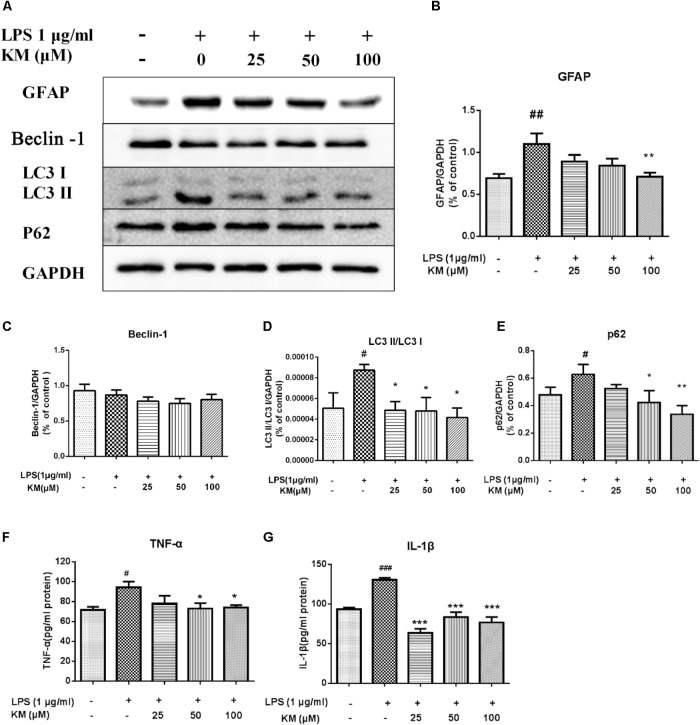
Koumine (KM) enhances LPS-induced astrocyte autophagy and attenuates astrocyte activation, as well as inflammation response in LPS-exposed primary astrocytes. Cultured astrocytes were exposed to koumine (0, 25, 50, or 100 μM) for 12 h prior to exposure to LPS for an additional 24 h before testing. Astrocytes in the control group were incubated with standard culture medium lacking koumine and LPS. Western blot **(A)** and densitometric quantification of GFAP **(B)**, Beclin 1 **(C)**, LC3-I/LC3-II **(D)**, P62 **(E)**, and GAPDH were performed in LPS-exposed astrocytes. Elisa were performed to explore the LPS-induced production of TNF-α **(F)** and IL-1β **(G)** in cultured astrocytes. Data are presented as the mean ± SEM. of three independent experiments. ^###^*P* < 0.001, ^##^*P* < 0.01, and ^#^*P* < 0.05 compared with the control group. ^∗∗∗^*P* < 0.001, ^∗∗^*P* < 0.01, and ^∗^*P* < 0.05 compared with the LPS group, as determined by one-way ANOVA followed by the LSD test.

To further confirm that koumine directly inhibited astrocyte reactivation and inflammation response, we performed Western blotting and ELISA testing in LPS-exposed rat primary astrocytes. First, we determined that several concentrations of koumine (0, 25, 50, and 100 μM) had no effect on cell viability at several concentrations after treatment for 24, 36, or 48 h by MTT assay (data not shown). Consequently, we pretreated the astrocytes with koumine (or vehicle) for 12 h and then stimulated them with LPS for 24 h to induce reactivation. In agreement with our previous reports, koumine, significantly reduced GFAP expression and the levels of IL-1β and TNF-α (**Figures 4A,B,F,G**). These results are consistent with our *in vivo* findings. In addition, we explored the effects of koumine on LPS-induced autophagy in primary astrocytes. We found the levels of Beclin-1 protein in astrocytes treated with LPS was not affected, the expression of p62 and the ratio of LC3-II/LC3-I increased significantly (*P <* 0.05), indicated LPS induces activation of astrocytes while activating autophagy. However, the increased expression of LC3-II/LC3-I and p62 protein induced by LPS could be inhibited by koumine (**Figures 4A,C,D,E**).

### Blockage of Autophagy Activity Diminished Analgesia Effect of Koumine

**FIGURE 5:**
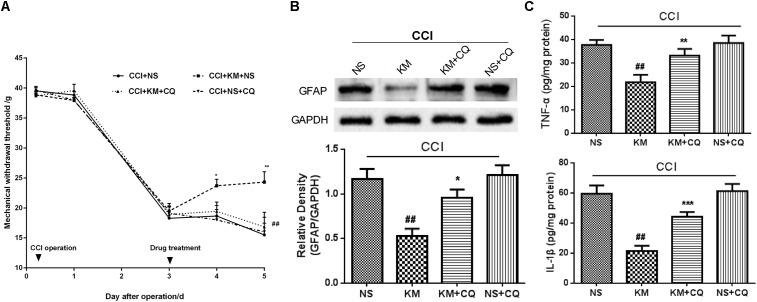
Blockage of autophagy activity diminished effect of koumine (KM) on MWT and astrocyte activation in CCI rats. **(A)** The antagonistic effect of chloroquine (CQ) against antinociceptive effect of koumine. Data indicate the withdrawal threshold for the ipsilateral paw as the mean ± SEM (*n* = 5–7 per group). ^∗^*P* < 0.05 and ^∗∗^*P* < 0.01 compared with the CCI+NS group. ^##^*P* < 0.01 compared with the versus CCI+KM+NS group, two-way repeated-measures ANOVA followed by LSD or Dunnett’s T3 test for each time point. **(B)** GFAP level was detected by Western blot in the spinal cord. **(C)** Pro-inflammatory cytokine IL-1β and TNF-α in spinal cord of CCI rats after the koumine (KM) and CQ administered. Data are expressed as the mean ± SEM. ^##^*P* < 0.01 versus CCI+NS group. ^∗^*P* < 0.05, ^∗∗^*P* < 0.01, and ^∗∗∗^*P* < 0.001 versus CCI+KM group, separate one-way ANOVA followed by LSD test.

To assess the pro-autophagic activity of koumine in the development of mechanical allodynia in CCI rats, we injected koumine subcutaneously for two consecutive days beginning on day 3 after the CCI operation, then autophagy inhibitor chloroquine (CQ) was administered. As shown in **Figure 5A**, in the CCI+KM group, repeat subcutaneous injection of koumine (7 mg/kg) significantly increased MWT (*P <* 0.05 for postoperative day 4, *P <* 0.01 for postoperative day 5). In the CCI+KM+CQ group, we selected the dose of CQ (0.1 μg, intrathecal injection) which did not affect the MWT. Interestingly, the increases in MWT caused by koumine were abolished by CQ (*P <* 0.1 for postoperative day 5). These data indicate that the analgesic effect of koumine was abolished by the autophagy inhibitor chloroquine.

### Blockage of Autophagy Activity Diminished the Effect of Koumine on Astrocyte Activation in CCI Rats

**FIGURE 6:**
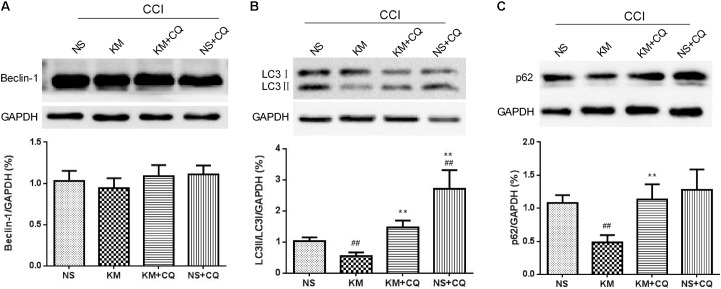
Koumine (KM) promoted autophagy in CCI rats. Beclin 1 **(A)**, LC3 **(B)**, and p62 **(C)** levels were detected by Western blot in the spinal cord. ^##^*P <* 0.05 versus Sham group. ^∗^*P <* 0.05 and ^∗∗^*P <* 0.01 versus vehicle control group by one-way ANOVA followed by LSD test.

We sought to further investigate whether the analgesic effect of koumine was linked to the inhibition of astrocyte activation via the promotion of autophagy in the spinal cord of CCI rats. We next detected GFAP and pro-inflammatory cytokine in the spinal cord of CCI rats after koumine and CQ were administered. As shown in **Figure 5B**, Western blot results showed that the levels of GFAP decreased after koumine treatment (*P* < 0.01, compared with the CCI+NS group), whereas the effect of koumine on inhibition of astrocyte activation was abolished by CQ (*P* < 0.05, compared to KM group). Although a slight increase in GFAP protein level in CQ alone group was recorded, it was non-significant (*P* > 0.05, compared to CCI+NS group). Similar observations were made for IL-1β and TNF-α, the pro-inflammatory cytokine closely related to pain. The ELISA results showed KM that significantly decreased IL-1β and TNF-α protein expression (*P* < 0.01, compared to CCI+NS group), but reversed by CQ (*P* < 0.01 for TNF-α, *P* < 0.001 for IL-1β, compared to CCI+KM group), no significant differences were observed in CQ and NS groups (**Figure 5C**). In addition, although the level of Beclin-1 has no remarkable changes compared to vehicle-treated CCI and CQ-treated CCI groups, it presented a decreasing trend (*P* < 0.05, **Figure 6**). LC3-II/I and p62 expression were decreased compared to vehicle-treated CCI group and CQ-treated CCI groups, suggesting that blockage of autophagy activity diminished the effect of koumine on astrocyte activation in CCI rats.

### Effects of Koumine on Apoptosis-Related Protein in CCI Rats

**FIGURE 7:**
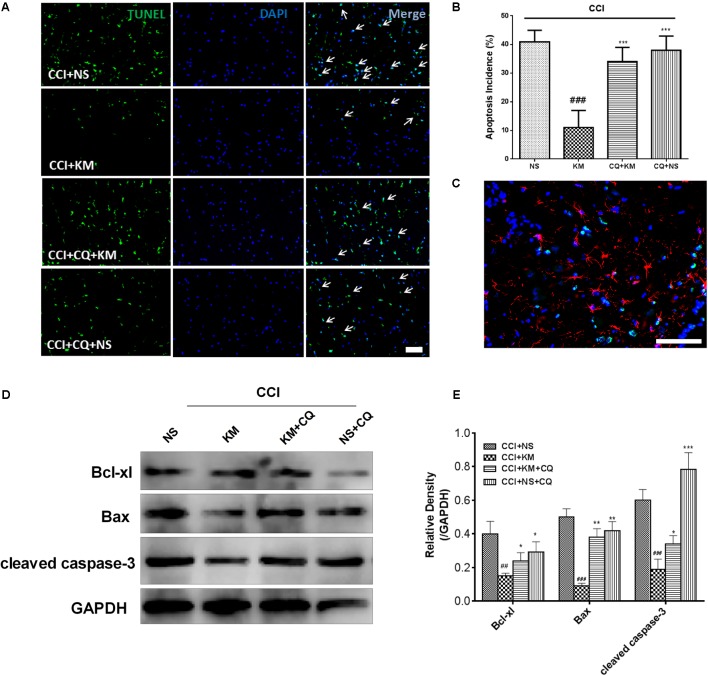
The effect of koumine (KM) on apoptosis in spinal cord tissue of CCI rats. **(A)** Apoptotic cells in rat spinal cord tissue through TUNEL staining. **(B)** Quantification of apoptotic cells in spinal cord tissue. **(C)** Representative immunofluorescence staining for glial fibrillary acidic protein (Red) and TUNEL-positive cells (green) merged double staining. Western Blot experiment **(D)** and the relative quantification **(E)** for apoptosis related protein Bcl-xl, cleaved caspase-3, and Bax protein expression in CCI rats with drug treatment. All results were obtained from three independent experiments. ^##^*P <* 0.01 and ^###^*P <* 0.001 versus CCI+NS group. ^∗^*P <* 0.05, ^∗∗^*P* < 0.01, and ^∗∗∗^*P* < 0.001 versus CCI+KM group by one-way ANOVA followed by LSD test.

Apoptosis was reported to be associated with the development of NP. We further investigated whether apoptosis was involved in the analgesic effects of koumine on CCI-induced NP. TUNEL staining of the rat spinal cord indicated that TUNEL-positive nuclei (co-immunostained TUNEL staining with DAPI) were present throughout the spinal cord, and their numbers were decreased by koumine (**Figure 7A**). Quantification of the apoptosis incidence (TUNEL- and DAPI-positive cells/DAPI-positive cells, **Figure 7B**) revealed a high occurrence (41%), which koumine treatment could decrease to 11% (*P* < 0.001, compared to CCI+NS group). Interestingly, this effect was abolished by CQ (*P <* 0.001, compared to CCI+KM group). Then, we costained GFAP with TUNEL in the spinal cord of CCI rats, the TUNEL staining almost invisible colocalization with GFAP positive glia cells (**Figure 7C**). Moreover, apoptosis related proteins were quantified by Western blot. The protein expression levels of cleaved caspase-3 and pro-apoptotic Bax protein were downregulated while anti-apoptotic Bcl-xl protein was upregulated after treatment with koumine (**Figures 7D,E**). Furthermore, CQ significantly decreased Bcl-xl and increased Bax and cleaved caspase-3 protein levels.

## Discussion

This research illustrated that analgesic effect of koumine, which might be modulated in part by attenuating astrocyte activation and the levels of pro-inflammatory cytokines via inducing autophagic flux and suppressing apoptosis in rats with CCI-induced NP.

The pathophysiological mechanism of NP is complex. However, an increasing amount of evidence suggests that neuroinflammation—characterized by activation of glia (including microglia and astrocytes) and pro-inflammatory cytokines—is an important factor in the development and maintenance of central sensitization and NP. Both activated microglia and astrocytes are participants in the NP and could be the main sources pro-inflammatory cytokines. Notably, microglia were activated at the early phase of the disease, whereas activated astrocytes were detected in the sustainment phase. The activation of astrocytes can release pro-inflammatory cytokines (such as TNF-α and IL-1β) and chemotactic factor, which, in turn, activate glia and neurons, eventually forming a positive feedback loop of glia-to-neuron signals, creating perseverative release of pain mediators. Astrocyte activation has been found in various injury conditions such as CCI, spinal nerve ligation (SNL), tissue injury, and inflammation, which are associated with enhanced pain states. Fluorocitrate, an astrocyte activation inhibitor, was able to relieve the mechanical pain in NP. Therefore, inhibition of astrocyte activation can be one of the new strategies to treat NP. In the present study, we investigate the astrocyte activation in CCI-induced NP rats. We found a significant activation of astrocytes, as shown by the increase in GFAP, which was decreased by treatment of koumine. In addition, TNF-α and IL-1β, the key pro-inflammatory pain mediators, were upregulated in the spinal dorsal horn following CCI and that koumine treatment markedly inhibited its productions. As activated astrocytes can be a source of TNF-α and IL-1β, these results imply that the analgesic effect of koumine may involve inhibiting astrocyte activation and pro-inflammatory cytokine production.

In recent years, the effect of autophagy in NP has attracted the attention of researchers. The impairment of autophagy occurred in NP was first described in the SNL mice and further confirmed in other experimental models of NP (Berliocchi et al., 2011, 2015). In turn, enhancing autophagy by pharmacological approaches has been reported to be a potential manner for slowing the onset and chronification of NP (Marinelli et al., 2014; Goldshmit et al., 2015). To investigate the correlation between autophagy and analgesic effect of koumine on NP, we assessed the changes in expression of LC3, Beclin1 and p62 in the spinal cord of CCI rats following treatment with koumine. After CCI operation, the rat displayed significant mechanical allodynia; meanwhile, the ratio of LC3-II/I and the expression of p62 increased significantly in the spinal cord, while Beclin-1 did not, suggesting that autophagic flux was blocked in the late stage of autophagy. In turn, administration of koumine significantly reversed mechanical allodynia, paralleled by decrease in LC3-II/I and p62, indicating that autophagy was enhanced by koumine. This evidence suggest the analgesic effect of koumine may be involved in enhancing autophagy in the spinal cord of NP rats. Indeed, several autophagy mediators were demonstrated to be effective in CNS diseases including NP. For example, simvastatin can contribute to neuroprotection after spinal cord injury by inducing autophagy (Gao et al., 2015). Rapamycin, an autophagy inducer, significantly attenuates NP by enhancing autophagy and inhibiting IL-1β expression in the microglia of the spinal cord (Feng et al., 2014). Ginsenoside compound K was reported to enhance autophagy in primary astrocytes by target of mTOR (mammalian target of rapamycin), which may promote β-amyloid peptide clearance and slow the pathological progression of AD (Guo et al., 2014). In contrast, blocking autophagy by pharmacological approaches can induce or enhance NP (Berliocchi et al., 2015). We found intrathecal injection CQ significantly diminishing the analgesic effect of koumine, which could also explain the analgesic activity of koumine on NP may be involved in promoting autophagy in rat spinal cord.

Although the precise mechanism of autophagy’s contribution to NP is not well understood, changes in autophagy flux in glial cells may play an important role. As koumine displayed strong inhibitory activity against astrocyte activation and promoting autophagy on spinal cord of rat. We further explored whether koumine could inhibit astrocyte activation by promoting astrocyte autophagy. Our studies demonstrated that LC3 staining showed a large number of colocalization with GFAP positive glia cells, suggesting that autophagy may occur in astrocytes of the spinal cord. Koumine treatment decreases the number of LC3/GFAP positive cells, indicating that koumine inhibits astrocyte activation and promotes astrocyte autophagy. On treatment with the autophagy inhibitor CQ, the inhibition effect of koumine on astrocyte activation was attenuated, along with the key pro-inflammatory molecules TNF-α and IL-1β. In cultured rat primary astrocytes, we found that koumine significantly decreased the upregulation of LC3II/LC3I and p62 protein induced by LPS, indicated koumine can promote the degradation of autophagic bodies and maintain the smooth flow of autophagy in astrocytes. Taken together, these results suggest that koumine may inhibit astrocyte activation by enhancing autophagy.

Autophagy is closely associated with apoptosis in many diseases, including NP, neurodegenerative diseases, and cancer. As a form of programmed cell death, apoptosis was reported to be associated with the development of NP (Amin et al., 2016; Zheng et al., 2017). Caspases and Bcl-2 family proteins, including the pro-apoptosis protein Bax and the pro-survival protein Bcl-xl, play a pivotal role in cell survival and death (Cheng et al., 1997). Interestingly, koumine was reported to serve as a protective effect against LPS-induced apoptosis on RAW 264.7 cells. In the present study, we demonstrated that the CCI model induced caspase-dependent apoptosis by significantly increasing Bax and cleaved caspase-3 and decreasing Bcl-xl expression. Koumine shown inhibit apoptosis and protective properties and may be a therapeutic agent for treating NP in CCI rats. However, we costained for GFAP and TUNEL in the rat spinal cord and revealed that unusual apoptosis occurred in astrocytes. It was reported that positive costaining of TUNEL and Nissl body cells indicates that a majority of apoptotic cells are neurons in NP animals. In addition, TNF-α and IL-1β, which are well known to take part in the regulation of cellular apoptosis (Hu et al., 2015). As we have demonstrated koumine significantly reduced the TNF-α and IL-1β expression in the spinal cord, we deduced that the main source of these apoptosis cells are neurons, while this needs to be further investigated.

## Conclusion

**FIGURE 8:**
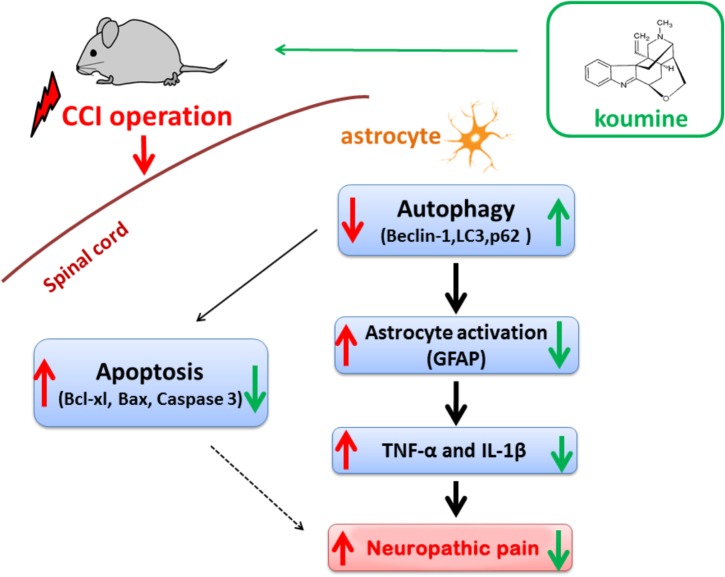
Proposed mechanisms of the effect of koumine in CCI rats. Koumine attenuates neuropathic pain through promoting astrocyte autophagy, inhibiting astrocyte excessive activation, decreasing astrocyte-mediated neuroinflammation and apoptosis.

The present study showed that autophagy was impaired in CCI-induced NP rats. Koumine treatment significantly enhanced autophagy and inhibited apoptosis and astrocyte activation as well as IL-1β and TNF-α production in the spinal cord, and the treatment also ameliorated CCI-induced mechanical allodynia (**Figure 8**). In the long term, koumine may provide useful innovative therapeutic strategies for the treatment of NP in clinical practice.

## Author Contributions

G-lJ and R-cY performed the experiments, analyzed the data, prepared the figures, and drafted the manuscript. S-dH and L-mH contributed to the experiments. YX analyzed the data and prepared the figures. C-xY conceived and designed the study. All authors have read and approved the final manuscript.

## Conflict of Interest Statement

The authors declare that the research was conducted in the absence of any commercial or financial relationships that could be construed as a potential conflict of interest.
